# The association between sexual orientation and psychotic like experiences during adolescence: a prospective cohort study

**DOI:** 10.1007/s00127-024-02636-y

**Published:** 2024-05-21

**Authors:** Emma Corcoran, Amal Althobaiti, Glyn Lewis, Francesca Solmi, Tayla McCloud, Gemma Lewis

**Affiliations:** 1 The Oxford Institute of Clinical Psychology Training and Research, The Oxford Centre for Psychological Health, Isis Education Centre, Warneford Hospital, Oxford, OX3 7JX UK; 2 Division of Psychiatry, University College London, Maple House, 149 Tottenham Court Road, London, W17 7NF UK

**Keywords:** Sexual orientation, Psychotic experiences, Adolescence, ALSPAC

## Abstract

**Purpose:**

Psychotic like experiences (PLEs) are relatively common during adolescence and associated with a range of negative outcomes. There is evidence that sexual minorities are at increased risk of mental health problems including depression, anxiety, self-harm and suicidality. However, no study has investigated the association between sexual orientation and psychotic experiences during adolescence. We compared trajectories of PLEs in sexual minority and heterosexual adolescents from 12 to 24 years of age.

**Methods:**

We used data from the Avon Longitudinal Study of Parents and Children (ALSPAC). Participants provided data on sexual orientation at age 16 and PLEs at ages 12, 17 and 24. We used multi-level logistic regression models to test associations between sexual orientation and PLEs, before and after adjusting for covariates. We investigated whether the association differed according to time-point and sex using interaction terms.

**Results:**

We found evidence that the odds of PLEs were 2.35 times (95% Confidence Interval 1.79–3.06, p < 0.0001) higher among sexual minority compared with heterosexual adolescents, across all ages, after adjusting for covariates. There was no evidence that the association between sexual orientation and PLEs differed according to time-point (p = 0.50) or sex (p = 0.29).

**Conclusion:**

We found an increased risk of psychosis in sexual minorities compared with heterosexuals, which was present from around 12 years of age and persisted until age 24. Early interventions to prevent this mental health inequality could include universal interventions to promote inclusivity and acceptance of diverse sexual orientations.

**Supplementary Information:**

The online version contains supplementary material available at 10.1007/s00127-024-02636-y.

## Introduction

Psychotic like experiences (PLEs) involve hallucinations and delusions (core symptoms of schizophrenia) without a diagnosis of psychotic disorder [1, 2]. PLEs are relatively common in the general population, particularly during childhood and adolescence [2]. Psychotic experiences are debilitating and increase the risk of subsequent psychotic disorders, depression, post-traumatic stress disorder, eating disorders and suicide [1–4]. These experiences have also been identified as markers of severe common mental disorder within the general population [5]. During adolescence, PLEs are associated with a range of other negative outcomes including lower educational attainment, substance use and criminal behaviour [6]. There is also evidence that persistent PLEs are associated with worse outcomes [7]. We have a relatively poor understanding of risk factors for psychotic experiences, which could inform interventions to prevent or reduce them. Adolescence, defined as 10 to 24 years of age [8], is particularly important for prevention as the peak age at onset for mental health problems is 14 and most mental health problems begin by age 25 [9].

Sexual minorities are often exposed to stigma, prejudice, discrimination and abuse within societies that promote being heterosexual and cisgender as normal. According to minority stress theory, these experiences create a hostile and stressful environment that causes mental health inequalities between sexual and gender minorities. There is evidence that the prevalence of depression, anxiety, self-harm and suicidality [10–14] is around twice as high in sexual minority compared with heterosexual adolescents [15]. However, to our knowledge, no study has investigated the association between sexual orientation and PLEs during adolescence. In adults, three cross-sectional studies [16–18] and one case–control study [19] find that psychotic disorders and symptoms are higher among sexual minorities than heterosexuals [20]. However, these findings are unlikely to generalise to adolescents who are experiencing a distinct developmental stage characterised by biopsychosocial transitions [8].

Another limitation of existing evidence is that the cross-sectional and case–control studies asked participants to recall psychotic experiences across their lifetime, which could lead to bias. This approach also does not provide evidence on when the increased risk of PLEs emerges, which could inform the timing of interventions. Longitudinal studies with repeated measures of mental health can be used to investigate when the risk of psychotic experiences emerges in sexual minority compared with heterosexual adolescents, and how it changes over time. Previous longitudinal studies suggest that depressive symptoms are higher in sexual minorities than heterosexuals from around 12 years of age [14, 21, 22], and this inequality persists throughout adolescence [14]. In this study, we investigated the association between sexual orientation and PLEs at multiple time-points during adolescence, using data from a population-based birth cohort study. We also estimated the population attributable fraction (PAF); the proportion of cases of psychotic symptoms that might be attributable to sexual-minority status if this relation were causal [23]. The PAF indicates the reduction in incidence that would occur if risks associated with the exposure were removed and is relevant to informing public health decisions.

## Methods

### Sample

**Fig. 1:**
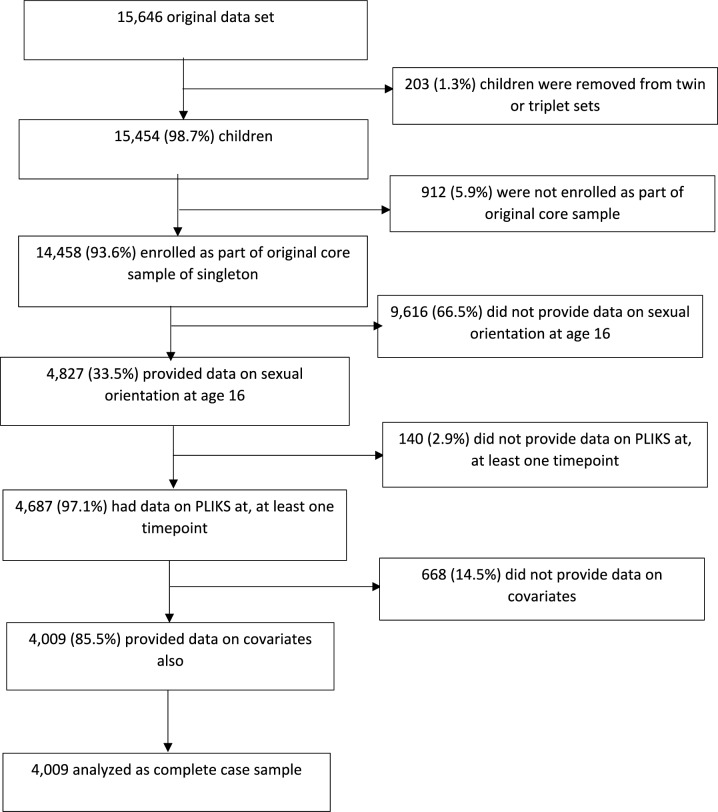
Flow chart of ALSPAC study participants. *ALSPAC* avon longitudinal study of parents and children, *PLIKS* psychotic experiences

We used data from the Avon Longitudinal Study of Parents and Children (ALSPAC). The original sample consisted of 15,454 pregnant women in the former county of Avon in Bristol, United Kingdom, with due dates between 1 April 1991 and 31 December 1992 [24–26]. We used data from core singleton offspring (excluding multiple births) who answered a question about sexual orientation when they were an average age of 16 (n = 4827, Fig. 1) [14–27].

The authors assert that all procedures contributing to this work comply with the ethical standard of the relevant national and institutional committees on human experimentation and with the Helsinki Declaration of 1975, as revised in 2008. Ethical approval for the study was obtained from the ALSPAC Ethics and Law Committee and the Local Research Ethics Committees. The study website (http://www.bristol.ac.uk/alspac/researchers/our-data/) contains details of all the data that is available through a fully searchable data dictionary and variable search tool. Study data were collected and managed using REDCap electronic data capture tools hosted at the University of Bristol.1 REDCap (Research Electronic Data Capture) is a secure, web-based software platform designed to support data capture for research studies [28]: https://projectredcap.org/resources/citations/.

### Procedure

#### Outcome—psychotic-like experiences (PLEs)

PLEs were measured at ages 12, 17 and 24 using the psychosis-like symptoms interview (PLIKSi). We included PLEs at age 12 because prior studies have found that differences in the mental health of sexual minorities and heterosexuals emerge early in adolescence, from as young as 11 years of age [29, 30]. The PLIKSi is a semi-structured interview consisting of 12 questions from the psychosis section of the schedule for affective disorders and Schizophrenia for school-age children (K-SDAS) [31] and the diagnostic interview schedule for children (DISC-IV) [32]. The interview explored the past 6-month presence of hallucinations, delusions and thought interference, with each symptom rated as not present, suspected, or definitely present. If a symptom was scored as suspected or definitely present, further questions were asked about the frequency and specificity of symptoms (i.e. location of voices, types of delusions [33]. If participants were able to give specific examples of the symptoms, they were marked as definite (unless they were related to substance use, lack of sleep or fever). We classified PLEs as a binary outcome [34], indicating the presence or absence of at least one suspected/definite symptom.

#### Exposure—sexual orientation

Sexual orientation was measured at a research clinic when participants were on average 16 years old. Participants were asked to choose the description that best fit how they thought about themselves. Response options were 100% heterosexual (straight), mostly heterosexual but also attracted to the same sex, bisexual (attracted to both sexes), mostly homosexual but also attracted to the opposite sex, 100% homosexual (gay), not sexually attracted to either sex and not sure. Consistent with other studies [11, 14, 27] and due to relatively small numbers in all responses except 100% heterosexual, we combined all non-heterosexual respondents into a single sexual minority category. We classified as sexual minority participants who were mostly heterosexual, bisexual, mostly homosexual, 100% homosexual and not sure [35]. This is consistent with previous evidence that all sexual minority groups, including those who identify as mostly heterosexual or not sure, have an increased risk of mental health problems compared with those identifying as 100% heterosexual [15]. In line with previous studies [14], individuals who were not attracted to either sex were excluded.

#### Covariates

Consistent with prior studies [12, 14] we adjusted for sex, maternal education (split into compulsory and non-compulsory [36]), maternal social class (split into manual and non-manual [37]). We used maternal data for these covariates because mothers were the unit of sampling in ALSPAC and there are substantial amounts of missing paternal data. In a posthoc sensitivity analysis, we further adjusted for maternal depressive symptoms assessed with the Edinburgh Postnatal Depression Scale; EPDS).

### Statistical analysis

All analyses were conducted in Stata version 15 [41]. Participants were included in the main analyses if they had complete data on the exposure and covariates, and at least one measurement of the outcome. First, we explored the characteristics of our sample according to sexual orientation. We then compared socio-demographic characteristics of participants with complete and missing data.

We used multilevel logistic regression models to investigate the association between sexual orientation and PLEs (Stata command ‘melogit’ with option ‘or’ to report odds ratios). Time-point was clustered within individual, with a random intercept at the level of the individual. We investigated whether the association between sexual orientation and psychotic like symptoms differed across time points, by calculating an interaction between sexual orientation and time (entered as a three-level categorical variable). We investigated whether the association between sexual orientation and PLEs differed by sex, by calculating an interaction between sexual orientation and sex. We examined associations with the three-category PLEs outcome using multinomial logistic regression (Stata command ‘mlogit’ with the ‘RRR’ option to report estimates relative risk ratios).

All models were run before and after adjusting for covariates. We calculated the population attributable fraction using the *punaf* command in Stata [14].

We conducted a sensitivity analysis using multiple imputation with chained equations (MICE), to explore the potential role of missing data. We started with the sample that had provided complete data on the exposure. We then imputed missing data in the outcome and covariates. We replaced missing data in outcome and covariates using multiple imputation with chained equations. We used the Stata command ‘mi estimate’ and imputed 25 datasets. Estimates were combined across datasets using Rubin’s rules. To predict missing data we used all variables in the main analysis, plus auxiliary variables: the Mood and Feeling Questionnaire (MFQ; ages 10 years, 12 years, 13 years, 17 years, 18 years, 22 years, 22 years, 11 months and 23 years) [42], IQ (measured at age 15 using the Wechsler Abbreviated Scale of Intelligence [43]), child anxiety scores (measured at age 24 using the Clinical Interview Schedule-Revised [44]) maternal depression (measured using the Edinburgh Postnatal Depression Scale [45]) maternal alcohol use during the first three months and last two months of pregnancy and maternal smoking during pregnancy. We selected auxiliary variables that were associated with missing data, based on existing evidence and theoretical assumptions. We estimated these associations using logistic regressions with ‘missing or not missing’ as the outcome and potential auxiliary variables as exposures. To improve prediction of missing outcome data, we restricted the sample with imputed data to those with at least one MFQ (in addition to having complete exposure data) [42].

### Supplementary analysis

As a posthoc supplemental analysis, we investigated whether participants identifying as sexual minority had increased risk of reporting psychotic experiences at multiple time points, since persistence of psychotic experience is a strong predictor of poorer clinical outcomes. For these analyses, we categorised participants according to the number of follow-up time-points at which they reported reported psychotic experiences (range none to three) and used multinomial logistic regressions (Stata command ‘mlogit’). We estimated Relative Risk Ratios (RRR), using the Stata option ‘rrr’. We ran both univariable and multivariable models, in the latter adjusting for the same covariates included in the main analyses. As in the main analyses, we ran these models both on a sample of participants with complete data and on participants who had complete exposure data, imputing covariate and outcome data.

## Results

### Sample

Of the 4827 participants with complete exposure data, 4687 (97.1%) provided outcome data on at least one time point. Of these, 4009 (85.5%) had complete data on all covariates and were included in our analytic sample (Fig. 1). In this sample, 516 participants (12.9%) identified as sexual minority. Sexual minority adolescents were more likely to be female and from families with higher education and social class levels (Table 1). Compared to participants with complete data, participants with missing data were more likely to be male, to have mothers with compulsory education and to report slightly more PLEs (Table 2). Among those with complete exposure there were no differences in the distribution of outcome and exposure between participants with complete or missing covariate data (Supplemental Table 1).

**Table 1:** Characteristics of the sample with complete data^a^ (n = 4009) according to sexual orientation

Characteristic – N (%)	Heterosexual (n = 3493)	Sexual minority (n = 516)	p value^b^
Female	1797 (51.5%)	339 (65.7%)	< 0.0001
Manual maternal social class^c^	500 (14.3%)	83 (16.1%)	0.287
Compulsory maternal education^d^	1706 (48.8%)	274 (53.1%)	0.071

^a^Participants with complete data on the exposure and covariates and at least one measure of psychotic experiences

^b^P values obtained from Chi-square tests

^c^Occupations dichotomised into manual and non-manual to form a social class measure based on the 1991 classification of the UK Office of Population Censuses and Surveys

^d^No A-Level or Degree qualification

**Table 2:** Characteristics of participants with complete data compared to those with missing data

Characteristic	Proportion of sample with missing data		Missing data sample^1^ (n = 9,779)	Complete case sample^2^ (n = 4,009)	p value^3^
	Overall	Among exposed			
	%	%	n (%)	n (%)	
Sexual Orientation	65.0%	–			
Sexual minority			109 (13.3%)	516 (12.9%)	
Heterosexual			709 (86.7%)	3493 (87.1%)	0.724
Psychotic experiences at age 12	53.9%	7.9%			
None			2227 (88.0%)	3398 (88.8%)	
Suspected/definite symptoms			305 (12.0%)	428 (11.2%)	0.294
Psychotic experiences at age 17	68.1%	27.8%			
None			1232 (89.9%)	2810 (93.0%)	
Suspected/definite symptoms			139 (10.1%)	212 (7.0%)	< 0.0001
Psychotic experiences at age 24	74.2%	44.8%			
None			1069 (85.7%)	2044 (88.3%)	
Suspected/definite symptoms			178 (14.3%)	271 (11.7%)	0.028
Sex	0%	0%			
Male			5242 (53.6%)	1873 (46.7%)	
Female			4,537 (46.4%)	2136 (53.3%)	< 0.0001
Maternal social class	28.0%	14.6%			
Manual			1392 (23.5%)	583 (14.5%)	
Non-manual			4,529 (76.5%)	3426 (85.5%)	< 0.0001
Maternal education	11.1%	2.9%			
Compulsory			2353 (28.6%)	1980 (49.4%)	
Higher			5890 (71.4%)	2029 (50.6%)	< 0.0001

^1^Participants with missing data on exposure, outcome and/or covariates

^2^Participants with complete data on the exposure and covariates and at least one measure of psychotic experiences

^3^P values obtained using chi-square tests to test the association of missing data with all variables included in our analysis

### Sexual orientation and PLEs

The prevalence of PLEs in the sample overall was 11.2% (n = 428) at age 12, 7.0% (n = 212) at age 17 and 11.7% (n = 271) at age 24 (Table 3). The prevalence of psychotic like symptoms was higher in sexual minorities compared with heterosexuals at each time-point (Table 3). In the univariable model, there was strong evidence that the odds of PLEs were higher in sexual minority compared with heterosexual adolescents (OR = 2.31, 95% CI = 1.78–3.02, p < 0.0001). After adjusting for covariates, this association remained similar (OR = 2.35, 95% CI = 1.79–3.06, p < 0.0001) (Table 3). There was no evidence that the association between sexual orientation and PLEs differed across time (p value for interaction = 0.50) or between sexes (p value for interaction = 0.29).

**Table 3:** Multilevel logistic regression models for associations between sexual orientation at age 16 and psychotic experiences, using sample with complete data^1^ (n = 4009)

Prevalence of psychotic experiences – N (%)^a^			
Psychotic experiences	Age 12	Age 17	Age 24
Heterosexual	346/3337 (10.3%)	162/2639 (6.1%)	209/1988 (10.5%)
Sexual minority	82/489 (16.8%)	50/383 (13.0%)	62/327 (18.9%)
Odds ratios (95% confidence intervals) at individual time-points^b^			
Psychotic experiences	Age 12 (n = 3826)	Age 17 (n = 3022)	Age 24 (n = 2315)
Unadjusted model	1.74 (1.34–2.26)	2.30 (1.64–3.22)	2.00 (1.46–2.72)
Adjusted model^c^	1.75 (1.34–2.28)	2.30 (1.64–3.25)	2.06 (1.50–2.82)
Overall odds ratios across time points			
Unadjusted model (n = 4009)	2.31 (1.78–3.02), P < .0001		
Adjusted model^b^ (n = 4009)	2.35 (1.79–3.06), P < .0001		

^a^Participants with complete data on exposure and confounders and at least one measure of psychotic experiences

^b^P values not reported for individual time-points because p values from sub-group analyses can be unreliable

^c^Adjusted for sex, maternal social class and maternal education

The population attributable fraction was 11.2% at age 12 (95% CI = 9.4–11.5%), 6.1% at age 17 (95% CI = 5.3–7.1%) and 10.5% at age 24 (95% CI = 9.2–11.9%) (Table 4). The PAF depends upon the effect size and the prevalence of the outcome amongst those exposed, which was lower at ages 17 than 12 and 24 (Table 3).

**Table 4:** Population attributable fractions^a^ with 95% confidence intervals, at each time-point

Age	Population attributable fraction
12	11.2% (9.4–11.5%)
17	6.1% (5.3–7.1%)
24	10.5% (9.2–11.9%)

^a^The proportion by which psychotic experiences would be reduced if all factors linking sexual orientation to psychotic experiences were addressed. [65]

### Sensitivity analyses

Results from the multiply imputed sample were similar to those from the sample with complete data (Table 5). There was no evidence that the association between sexual orientation and PLEs varied across time-point or by sex. Results were unaltered after adjustment for maternal depressive symptoms (Supplemental Table 2).

**Table 5:** Multilevel logistic regression models for associations between sexual orientation at age 16 and psychotic experiences, using imputed data (n = 4842)

Psychotic experiences	Odds ratios across all time points
Unadjusted model	2.30 (1.81–2.93), P < 0.0001
Adjusted model^a^	2.33 (1.84–2.93), P < 0.0001

^a^Adjusted for sex, maternal social class and maternal education

### Supplemental analyses

Results are shown in supplemental Table 3 and 4. In participants with complete data, we found evidence that sexual minority participants had an increased risk compared to heterosexual participants of PLEs at one (multivariable relative risk ratio [mRRR]: 1.61, 95% CI 1.14–2.26, p < 0.0001), two (mRRR: 3.64, 95% CI 2.21–6.01, p < 0.0001) and three (mRRR: 4.81, 95% CIs 2.04–11.35, p < 0.0001) time points in a dose–response fashion. Sexual minority participants were also at higher risk of reporting psychotic experiences at all three time points (mRRR: 4.81, 95% CIs 2.04–11.35, p < 0.0001) although for this association confidence intervals were wide—possibly due to fewer participants reporting the outcome at this frequency—and overlapping with those of the estimates for two time points, so inferences about dose–response patterns are difficult to make. In analyses conducted in the imputed sample, results were comparable, although here confidence intervals were narrower—due to the increased sample size.

## Discussion

In this large population-based birth cohort, we found evidence that sexual minority adolescents were more likely to experience PLEs than heterosexuals. The association between sexual orientation and PLEs was observed at age 12, and persisted over time (from age 12 to 24). There was no evidence that the magnitude of the association between sexual orientation and PLEs changed with age. We also found evidence that sexual orientation was associated with increasing number of PLEs in a dose–response fashion. Our findings suggest that the risk of PLEs is already higher in sexual minorities compared with heterosexuals during early adolescence, representing a mental health inequality that persists to adulthood. We also found evidence that the incidence of adolescent PLEs could be reduced by 11.2% at age 12, 6.1% at age 17 and 10.5% at age 24, if the risks associated with sexual minority orientation were removed.

### Strengths and limitations

To our knowledge, this is the first study of sexual orientation and PLEs during adolescence; a critical time for the onset and prevention of mental health problems. We measured PLEs at multiple time-points during adolescence, which enables us to investigate changes in the association with sexual orientation. Although our results suggest that sexual minority orientation is associated with increased risk of PLEs at single and multiple time-points, we did not separate individuals with single versus persistent episodes. Our prospective design was a strength as it minimised recall bias. We also used a semi-structured interview based on principles of standardized clinical examination to measure PLEs. This should reduce biases that might be introduced by self-reports.

Missing data is a limitation of all cohort studies and complete case analyses can lead to bias. However, our results were unaltered after we replaced missing data using multiple imputation, suggesting that missing data are unlikely to have biased our associations. We assumed missingness was dependent on observed data (missing at random). Although we cannot be certain that data were missing at random, the large amount of data in ALSPAC allowed us to identify several variables associated with missingness, supporting the plausibility of the assumption. We cannot exclude the possibility that data were missing not at random, which could lead to biases not corrected by multiple imputation. For example, if participants with more severe psychotic symptoms were more likely to drop out over time, and this was not corrected in imputations, it could have reduced the size of our associations or accounted for the decline in the association over time.

In cohort studies with long follow-up periods, the main source of missing data is usually the outcome, due to attrition. Imputation models that replace data in the variables with the most missing data, including the outcome, have been found to be the least biased in ALSPAC [46]. ALSPAC also contains a wealth of data to predict the outcome. When auxiliary variables are added, prediction of missing outcomes is further strengthened and risk of bias reduced. We therefore think the approach of imputing the outcome is justified. Another limitation of our study was that, due to attrition, our analyses were based on a sub-sample of the initial ALSPAC cohort, which could lead to selection bias. ALSPAC also has a high proportion of white participants and we were not able to include ethnicity in our analyses. As there is an association between ethnicity and PLEs [47], it would be useful for future studies to examine whether associations are influenced by ethnicity.

There were some limitations in how we measured and classified sexual orientation. We classified all participants who were not 100% heterosexual into one sexual minority group, due to small numbers in some sexual minority categories. This increased the power and precision of our analyses. The added rationale was evidence that all sexual minority groups are at increased risk of mental health problems compared with heterosexuals [29]. However, there is evidence that certain sexual minority groups are at increased risk of mental health outcomes compared with others [48]. Bisexual women, for example, have been found to have higher rates of depression than other sexual minority groups [15, 48–50]. Further research in larger samples would be useful, to investigate whether associations between sexual orientation and PLEs differ according to sexual minority group and gender. Eighty nine percent of participants who attended the research clinic at age 16 provided data on their sexual orientation. It is possible that participants who provided data on their sexual orientation differed systematically to those who did not, leading to selection bias. Sexual orientation may have been under-reported due to perceived stigma. However, if the heterosexual group contained sexual minority adolescents who had higher PLEs, or if some sexual minority adolescents chose not to report their sexuality, this misclassification is likely to have attenuated our associations, rather than introduced a spurious effect [14].

Our assessment of sexual orientation occurred after one of our psychotic experience outcomes (at 12 years of age). Many adolescents who identified as sexual minority at 16 years of age would not have been fully aware of their emerging sexual orientation at age 12. However, the development of sexual orientation is a multifaceted process that usually unfolds over many years [51]. A recent meta-analysis found that LGBQ + people first became aware of attraction to the same gender at an average age of 12.7, though there was of course substantial heterogeneity. LGBQ + people first questioned their sexual orientation at an average age of 13.2. [51] It is also possible that people who become non-heterosexual are perceived differently by others from a young age and this affects mental health. Longitudinal studies have shown that young people who identified as sexual minority at age 16 were more likely to be bullied from the age of 10 [52]. There is also evidence of a prospective association between gender non-conformity during childhood and sexual minority identification during adolescence [53, 54].

We measured sexual orientation at one point in time whereas sexual orientation is often fluid [55], particularly among young people. Our results suggest that identifying as sexual minority at age 16 is associated with mental health problems but future studies could investigate how fluidity in sexual orientation might affect mental health [56]. The use of a single question about sexual attraction and identity might have classified fewer individuals as sexual minority than would separate questions on attraction, behaviour, and identity. Finally, ALSPAC used binary measures of gender and we were unable to identify trans (transgender, non-binary or gender diverse) adolescents. Large high-quality population-based studies of the mental health of trans adolescents are needed [57].

### Mechanisms

Sexual minorities experience unique stressors due to stigma, discrimination and prejudice within societies that predominantly treat heterosexual orientations as normative [58]. Compared with heterosexuals, sexual minority adolescents are more likely to experience bullying [12, 38, 39], which is associated with an increased risk of PLEs [40]. Family conflict and feelings of shame, guilt, isolation, loneliness and rejection are also likely to contribute to the increased risk of PLEs in sexual minority adolescents compared with heterosexuals [14, 59].

Our aim was to conduct the first study of the association between sexual orientation and PLEs among adolescents. Future studies could investigate the mechanisms underlying this association, to inform the development of interventions.

### Implications

Our findings provide strong evidence of mental health inequalities between sexual minorities and heterosexuals, from early in adolescence [21, 60]. We found evidence of an increased risk of PLEs in sexual minority young people compared with heterosexuals at 12 years of age. This suggests that universal interventions to prevent PLEs should occur earlier than this, in childhood. Schools are a potential setting for universal interventions that would reach most adolescents. Promoting inclusivity and acceptance of diverse sexual orientations in schools could improve the mental health of sexual minority adolescents. Inclusive anti-bullying policies and curriculums and gay-straight alliances have been found to improve the school environment and the mental health of students [61–63]. It is important to acknowledge that sexual minority adolescents are living in a largely heteronormative society, which may make lead to isolation, marginalisation and mental health difficulties [29]. Despite some initial attempts to introduce a more diversified curriculum, the main focus remains on heterosexual relationships within most schools. Education could incorporate wider social issues and influences on the attitudes and behaviours of adolescents by challenging these heteronormative assumptions, rather than focusing on isolated issues of bullying [14, 64]. Fostering safety and inclusivity for sexual minority adolescents is a responsibility that everyone must take in order to protect these individuals from PLEs and other associated mental health and social difficulties.

## Supplementary Information

Below is the link to the electronic supplementary material.

Supplementary file1 (DOCX 22 KB)

## Data Availability

ALSPAC data can be accessed by researchers, upon request: https://www.bristol.ac.uk/alspac/researchers/access/.
