# Supplementary material for: The association between sexual orientation and psychotic like experiences during adolescence: a prospective cohort study
Source: Soc Psychiatry Psychiatr Epidemiol. 2024 May 21;59(12):2351–60. doi: 10.1007/s00127-024-02636-y (PMC11522136; doi:10.1007/s00127-024-02636-y)
Supplement: Supplementary file 1 — Supplementary file1 (DOCX 22 KB) [file 127_2024_2636_MOESM1_ESM.docx]

Supplemental Table 1: Distribution of outcomes and exposure among those with exposure and complete or missing covariates n=4,827

| **Participant characteristic** | **Participants with exposure available, n=4,827** | | |
| --- | --- | --- | --- |
|  | **At least one missing covariate data** | **Complete**  **covariate data** | **p-value** |
| **Total** | 712 (14.7%) | 4,115 (85.3%) |  |
| **Sexual orientation** |  |  |  |
| Heterosexual | 625 (14.9%) | 3,577 (85.1%) | 0.530 |
| Sexual Minority | 87 (13.9%) | 538 (86.1%) |  |
| **Psychotic like experiences at age 12** |  |  |  |
| None | 551 (13.9%) | 3,398 (85.1%) | 0.883 |
| Suspected/definite | 68 (13.7%) | 428 (86.3%) |  |
| **Psychotic like experiences at age 17** |  |  |  |
| None | 423 (13.1%) | 2,810 (86.9%) | 0.341 |
| Suspected/definite | 38 (15.2%) | 212 (84.8%) |  |
| **Psychotic like experiences at age 24** |  |  |  |
| None | 309 (13.1%) | 2,044 (86.8%) | 0.894 |
| Suspected/definite | 40 (12.9%) | 271 (87.1%) |  |

Abbreviations: number (n)

Supplemental Table 2: Multilevel logistic regression models for associations between sexual orientation at age 16 and psychotic like experiences, using sample with complete exposure and imputed confounders and outcomes (n=4,827).

| **Overall odds ratios (and 95% confidence intervals) of psychotic like experiences across time points** | |
| --- | --- |
| Unadjusted model | 2.34 (95% CI 1.82-3.03), p<.0001 |
| Adjusted model^1^ | 2.38 (95% CI 1.84-3.07), p<.0001 |
| Adjusted model^2^ | 2.37 (95% CI 1.84-3.07), p<0.0001 |

Abbreviations: number (n); confidence interval (CI); p-value (p)

^1^Adjusted for sex, social class and maternal education

^2^ Adjusted for sex, social class, maternal education, and maternal depressive symptoms in pregnancy

Supplemental Table 3. Distribution of number of time points with psychotic like experiences reported, according to sexual orientation.

| **Sexual orientation** | **Total number of follow-up points in which participants report psychotic like experiences** | | | |
| --- | --- | --- | --- | --- |
|  | None | One | Two | Three |
|  | n (%) | n (%) | n (%) | n (%) |
|  | Sample based on all available data (n=2,156) | | | |
| Heterosexual | 1,488 (80.1%) | 282 (15.2%) | 69 (3.7%) | 18 (1.0%) |
| Sexual minority | 199 (66.6%) | 63 (21.1%) | 27 (9.0%) | 10 (3.3%) |
|  | Sample based on complete cases (n=1,896) | | | |
| Heterosexual | 1,317 (80.4%) | 247 (15.1%) | 60 (3.7%) | 15 (0.9%) |
| Sexual minority | 171 (66.5%) | 51 (19.8%) | 26 (10.1%) | 9 (3.5%) |

Abbreviations: number (n)

Supplemental Table 4. Univariable and multivariable multinomial logistic regression models testing the association between sexual orientation and persistence of psychotic experiences.

| **Sexual orientation** | **Persistence of PEs** | | | | | |
| --- | --- | --- | --- | --- | --- | --- |
|  | One time point (vs. none) | | Two time points (vs. none) | | Three time points (vs. none) | |
|  | Univariable  RRR (95% CI)  p-value | Multivariable^1^  RRR (95% CI)  p-value | Univariable  RRR (95% CI)  p-value | Multivariable^1^  RRR (95% CI)  p-value | Univariable  RRR (95% CI)  p-value | Multivariable^1^  RRR (95% CI)  p-value |
|  | **Sample based on complete cases (n=1,896)** | | | | | |
| Heterosexual | Reference | Reference | Reference | Reference | Reference | Reference |
| Sexual minority | 1.59 (1.13 to 2.24)  P<0.0001 | 1.61 (1.14 to 2.26)  P=0.007 | 3.33 (2.05 to 5.43)  P<0.0001 | 3.64 (2.21 to 6.01)  P<0.0001 | 4.62 (1.99 to 10.72)  P<0.0001 | 4.81 (2.04 to 11.35)  P<0.0001 |
|  | **Sample based on imputed sample (n=4,827)** | | | | | |
| Heterosexual | Reference | Reference | Reference | Reference | Reference | Reference |
| Sexual minority | 1.62 (1.28 to 2.08)  P<0.0001 | 1.65 (1.30 to 2.11)  P<0.0001 | 2.69 (1.87 to 3.88)  P<0.0001 | 2.77 (1.92 to 4.00)  P<0.0001 | 3.11 (1.61 to 5.98)  P=0.001 | 3.11 (1.60 to 6.00)  P=0.001 |

Abbreviations: Relative Risk Ratio (RRR); Confidence Interval (CI); P-value (p)

^1^Adjusted for sex, social class and maternal education
